# Rationale and methods of the cardiometabolic valencian study (escarval-risk) for validation of risk scales in mediterranean patients with hypertension, diabetes or dyslipidemia

**DOI:** 10.1186/1471-2458-10-717

**Published:** 2010-11-22

**Authors:** Vicente Gil-Guillen, Domingo Orozco-Beltran, Josep Redon, Salvador Pita-Fernandez, Jorge Navarro-Pérez, Vicente Pallares, Francisco Valls, Carlos Fluixa, Antonio Fernandez, Jose M Martin-Moreno, Manuel Pascual-de-la-Torre, Jose L Trillo, Ramon Durazo-Arvizu, Richard Cooper, Marta Hermenegildo, Luis Rosado

**Affiliations:** 1 University Miguel Hernandez. Department of Clinical Medicine. San Juan de Alicante, Spain; 2 University of Valencia. Department of Medicine. Valencia, Spain; 3 University A Coruña. A Coruña, Spain; 4 Unión de Mutuas. Castellón, Spain; 5 Centro de Salud Beniganim. Valencia, Spain; 6 Centro de Salud Benimaclet. Valencia, Spain; 7 ESCARVAL Project. Valencia, Spain; 8 Director of Programme Management WHO Regional Office for Europe. Copenhagen, Denmark; 9 Department of Health; Valencia Government. Valencia, Spain; 10 Loyola University of Chicago. Stricht School of Medicine. Department of Preventive Medicine and Epidemiology. Chicago, USA

## Abstract

**Background:**

The Escarval-Risk study aims to validate cardiovascular risk scales in patients with hypertension, diabetes or dyslipidemia living in the Valencia Community, a European Mediterranean region, based on data from an electronic health recording system comparing predicted events with observed during 5 years follow-up study.

**Methods/Design:**

A cohort prospective 5 years follow-up study has been designed including 25000 patients with hypertension, diabetes and/or dyslipidemia attended in usual clinical practice. All information is registered in a unique electronic health recording system (ABUCASIS) that is the usual way to register clinical practice in the Valencian Health System (primary and secondary care). The system covers about 95% of population (near 5 million people). The system is linked with database of mortality register, hospital withdrawals, prescriptions and assurance databases in which each individual have a unique identification number. Diagnoses in clinical practice are always registered based on IDC-9. Occurrence of CV disease was the main outcomes of interest. Risk survival analysis methods will be applied to estimate the cumulative incidence of developing CV events over time.

**Discussion:**

The Escarval-Risk study will provide information to validate different cardiovascular risk scales in patients with hypertension, diabetes or dyslipidemia from a low risk Mediterranean Region, the Valencia Community.

## Background

Cardiovascular diseases (CVD) are the leading causes of mortality (13%) and one of the most important producing disability-associated living years (DALYs) (10%) in over de world. Population research and public health surveillance have made enormous contributions to the control of cardiovascular diseases (CVD) over the last 50 years. In virtually all industrialized countries the mortality from stroke and heart disease has declined 50-70%. Much of this success can be attributed to the identification of the underlying causes of CVD that are embedded in contemporary lifestyle. Despite the capacity of prevention, CVD are considered an epidemic in Europe with over 4.3 million deaths per year, accounting for the leading cause of death [1]. CVD mortality and its trends vary within European Regions and the Mediterranean area shows the lowest mortality rates [1]. In these countries, the growth and progressive ageing of the population in recent years have led to the paradoxical situation of reduced age-adjusted CHD mortality on the background of an increased number of deaths which tend to occur later in life and, consequently, a greater burden of disease and disability [2]. In Spain, a Mediterranean country, CVD is the leading cause of death accounting for 122793 deaths with a standardized rate of 231/106Inhab in 2008 [3-5]. The Valencia Community, a Spanish Mediterranean region located on the east-coast, with a population of 4885029, had CVD as the leading cause of death too, accounting for 13241 deaths with a standardized rate of 258/106Inhab in 2008, one of the highest rates in Spain [3].

There is a large and compelling body of evidence on the efficacy of primary CVD prevention [6], and of its population impact. Risk scales (Frammingham, Score) help clinicians to identify those patients to be selected for intervention. Global coronary heart disease (CHD) risk information given to patients seems to improve the accuracy of risk perception and may increase intent to initiate CHD prevention among individuals at moderate to high risk. The effect of global risk presentation on more remote outcomes is less clear and seems to be related to the intensity of accompanying interventions [7].

Scale uncertainties applying to different populations that those of their origin leads to the necessity of validation in different countries, regions or specific group of patients. In Spain this was done by using prevalence not incidence [8] and one calibration [9] have been made. Likewise, our group tried to validate the scales using incidences but with only one year of follow-up [10,11].

ABUCASIS, an electronic centralized clinical record system for primary and secondary ambulatory care was started in Valencia Community in 2003. The electronic health recording system (HER) of the whole population, around 5 millions of individuals is an excellent tool to initiate new studies about incidence of CVD and to validate the CHD scales widely used in clinical practice.

The aim of this study is to provide information to validate different cardiovascular risk scales and to analyze associated factors in patients with hypertension, diabetes or dyslipidemia from a low risk Mediterranean European Region. Incidence of cardiovascular events will be compared with expected events from the cardiovascular risk scales.

## Methods/Design

### Design

Escarval means *EStudio CARdiometabolico VALenciano*. The Escarval-Risk is a 5 years cohort (2007-2012) follow-up study. The study accounts with the support of Valencian Health Authorities, the universities of Valencia and Miguel Hernandez Elche, and the scientific societies of Valencian primary care physicians (*Sociedad Valenciana de Medicina de Familia i Comunitaria *and *Sociedad Española de Médicos de Atención Primaria Comunidad Valenciana*). This project has been included in the Valencia Cardiovascular Health Plan 2007-2012.

### Setting

The sample will be recruited from the population receiving healthcare from the Valencia Health Agency. The Valencia Community is a Spanish Mediterranean region located on the east-coast, with a population (year 2007) of 4,885,029, with 3,205,724 people older than 30 yr.

### Study participants

The study sample was selected in a two-step process that involved recruitment of clinicians and their outpatients. Around 800 clinicians have been selected to participate (family physicians and nurses) from the 23 Health Departments which have started with the ABUCASIS system. In order to stimulate involvement in ESCARVAL-RISK, the Valencia Health Agency have incorporated the participation in the study into the governance contract with the Health Departments, and linked it to the existing system of economic incentives associated to quality indexes.

**Figure 1:**
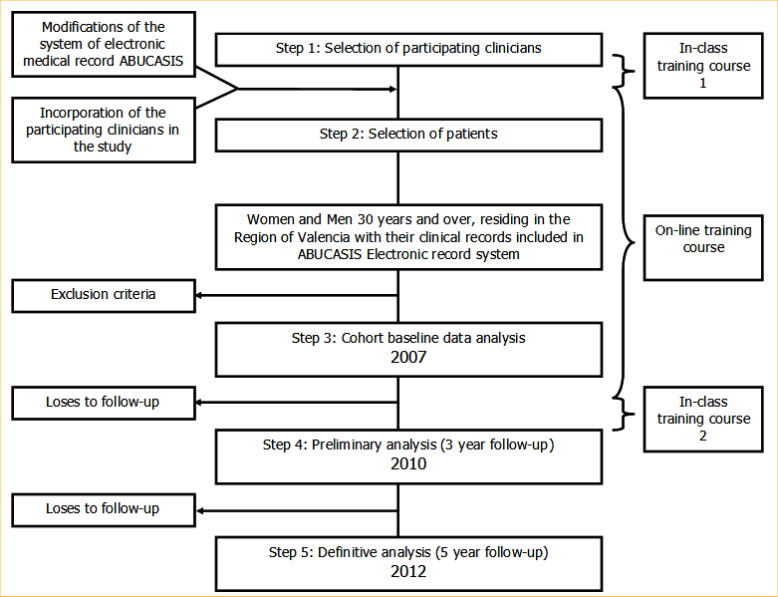
**Flow diagram of the Escarval-Risk study**.

Patients aged 30 years or older with at least one of the following CVD risk factors: hypertension, diabetes mellitus and/or dyslipidemia who attended for routine consultation with the participating clinicians have been selected (Table 1 and Figure 1). Patients with a history of a previous CVD event, that those defined as the outcomes (for ex myocardial infarction, angina, stroke, or transitory ischemic attack) were excluded, as well as patients already participating in clinical trials.

**Table 1:** Criteria to include patients in the ESCARVAL-RISK study.

1. Age 30 years or older	
2. Free of clinical cardiovascular disease	

3. At least one of the following cardiovascular risk factors (as assessed from the most recent data in the clinical record or anthropometry for obesity)	

a) Hypertension	- Systolic blood pressure ≥ 140 mm Hg, or
	- Diastolic blood pressure ≥ 90 mm Hg, or
	- Under antihypertensive medication

b) Diabetes mellitus	- Fasting plasma glucose ≥ 7.0 mmol/l (126 mg/dl), or
	- Under antidiabetic medication (insulin or oral medications)

c) Dyslipidaemia	- LDL cholesterol ≥ 4.1 mmol/l (160 mg/dl), or
	- HDL cholesterol < 1.036 mmol/l (40 mg/dl) in men, and < 1.30 mmol/l (50 mg/dl) in women, or
	- Triglycerides ≥ 1.7 mmol/l (150 mg/dl), or
	- Under lipid-lowering medication.

4. Informed consent	

This sample size would make it possible to detect a relative risk of ≥ 1.2, estimating an exposed proportion of 50% and a proportion of censured observations of 88.0%, with a security of 95% and a statistical power of 80%. The total study population will be 25,001 patients. Table 2 shows the participating patients by sex, and age. The percentage of participating patients among those invited was 80.1%.

**Table 2:** Participating patients by sex, and age.

Hypertension					
**Age Group**		**men**	**women**	**Total**	**%**

	30-39	216	145	361	2.91
	
	40-49	895	631	1526	12.31
	
	50-59	1554	1265	2819	22.74
	
	60-69	2126	1880	4006	32.32
	
	70-79	1356	1467	2823	22.77
	
	80 and more	350	511	861	6.95

	Total	6497	5899	12396	100.00

**Diabetes mellitus**					

Age Group		men	women	Total	%

	30-39	101	77	178	2.81
	
	40-49	465	230	695	10.96
	
	50-59	910	552	1462	23.06
	
	60-69	1295	808	2103	33.17
	
	70-79	796	661	1457	22.98
	
	80 and more	189	256	445	7.02
	
	Total	3756	2584	6340	100.00

**Dyslipidemia**					

Age Group		men	women	Total	%

	30-39	295	240	535	5.50
	
	40-49	906	601	1507	15.49
	
	50-59	1288	1168	2456	25.24
	
	60-69	1497	1468	2965	30.47
	
	70-79	816	1014	1830	18.81
	
	80 and more	153	284	437	4.49

	Total	4955	4775	9730	100.00

### Study variables and methods of data collection

**Figure 2:**
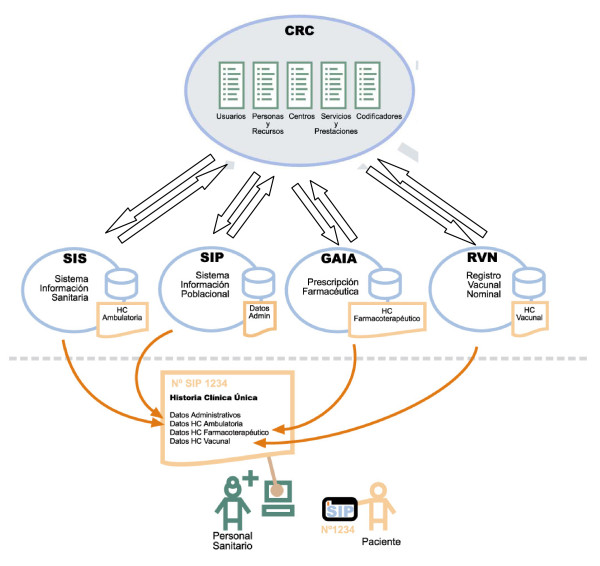
**Source of information for ESCARVAL-RISK study: ABUCASIS Clinical record system linked with health information databases**.

**Figure 3:**
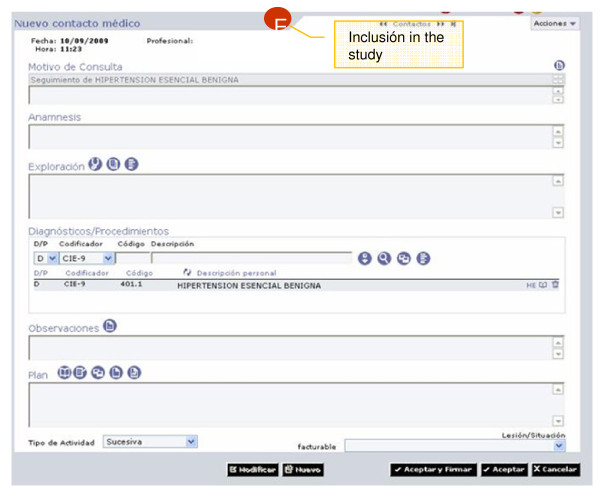
**Source of information for ESCARVAL-RISK study: ABUCASIS Clinical record system: A patient's record**.

**Figure 4:**
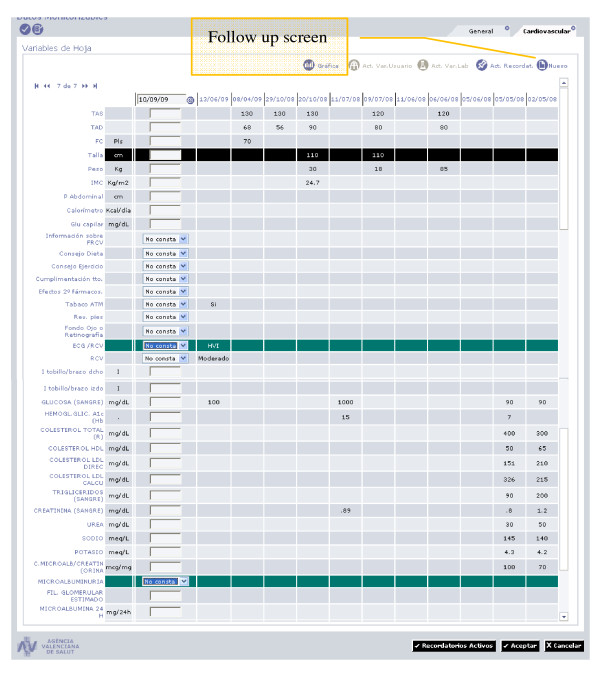
**Source of information for ESCARVAL-RISK study: ABUCASIS Clinical record system: Patient's follow up screen**.

Table 3 includes a description of data collection methods and variables in the ESCARVAL-RISK study. Information is collected prospectively from the ABUCASIS the only way to register patient's information. Diagnoses are registered by using IDC-9. To be sure of the validity of the principal variables (mortality, cardiovascular events), those other database resources are used. Every patient has a unique personal identification number (PIN) for the health system, so there is a unique electronic centralized clinical record *per *patient and the PIN allow to link other resources of data (Figures 2,3,4).

**Table 3:** Data collection methods and main study variables in ESCARVAL-RISK study.

Collection method	Variables	
Mortality register, Hospital dropouts database, electronic clinical record ICD diagnoses.	Cause of death (All, Cardiovascular or not) Cardiovascular Morbidity:	ICD-9 codes
	- Ischemic heart disease	402.9; 404.9, 410-414
	- Stroke.	430-438, 444
	- Peripheral Vascular Disease.	440.2, 443.8, 444, 445, 447.9
	- Aortic aneurysm.	441
	- Heart failure.	428, 398.91
	- Atrial fibrillation.	427.3
	- Chronic renal insufficiency.	585.5, 585.6, 588.88, 593.9 791.0
	- Proteinuria.	
	- Essential hypertension.	401, 403, 404, 405, 405.1, 405.11, 405.19, 997, 997.91, 401.0, 401.1, 401.9
	- Diabetes mellitus.	250.0, 250.4, 362.0, 583.81, 581.81, 250.6, 250.5
	- Hypercholesterolemia/hyperlipidemia.	272.0, 272.2
	- Metabolic syndrome.	277.7
	- Overweight and obesity.	278
	- Retinopathy.	362.0, 362.11, 362.12, 362.81, 362.82, 363.41, 362.83.

Electronic clinical record abstraction of patients' characteristics or measurements performed on patients during the medical visits	Patients' demographic and psychosocial characteristics	
	Relevant family medical history: early cardiovascular event	
	CVD risk factors related to lifestyle (tobacco smoking, physical activity)	
	Weight, height, waist and hip circumference, and blood pressure, under standardized conditions. Left ventricular hypertrophy (Cornell criteria). Microalbuminuria (30-300 mg/g). Comorbidity	
	- Current medication: antihypertensives, statins and other lipid-lowering drugs, oral	
	- antidiabetics, insulin, anticoagulants, aspirin, and combination drug therapy	

Laboratory results taken from the clinical record (most recent blood data and physical examination during the previous year)	Total cholesterol, LDL cholesterol, HDL cholesterol, non-HDL cholesterol, triglycerides, apolipoprotein B, blood pressure, glucose, hemoglobine-A1c, creatinine, glomerular filtration rate (MDRD), fibrinogen (mg/dl), uric acid.	

At least one fasting blood sample is obtained every year. Laboratory parameters are obtained from the Hospital where the clinician usually referred it. In a 10% random sample of all centers with participating physicians underwent a site visit for data monitoring and audit, to ensure data quality.

### Ethics

The study is conducted according to the standards of the International Guidelines for Ethical Review of Epidemiological Studies (Council for International Organizations of Medical Sciences-CIOMS-Geneva, 1991) and the recommendations of the Spanish Society of Epidemiology about the review of ethical aspects of epidemiological research. The ESCARVAL-RISK study has been reviewed and approved by the Committee for Ethics and Clinical Trials of the Center for Public Health Research (*Comite Ético de Investigación Clínica (CEIC) de la Dirección General de Salud Pública y Centro Superior de Investigacion de Salud Pública (DGSP-CSISP*)). The ESCARVAL-RISK is a naturalistic, observational study undertaken as part of routine clinical practice, with no special interventions (except a training program for participating clinicians) to the patients included in the study. No additional risks associated with participation are anticipated, as any additional diagnosis, evaluation or treatment will be provided, apart from what the attending physician deems appropriate.

#### Confidentiality of the data

All information relative to the patient's identity is considered confidential. Patients' data collected from the ABUCASIS during the study will be documented anonymously, making impossible to use this information to identify the patients. The only link between the data and the patient is a code used exclusively for this study, in such a way that only the ABUCASIS system will be able to associate the data to an identified or identifiable individual. The data generated during the study will be handled according the Law 5/1999 and corresponding norms. All of the researchers with access to the data used in the study will be required to sign a document guaranteeing confidentiality.

#### Informed consent

Although the study does not involve the randomization of the sample or the application of further interventions, prior to inclusion, all patients must read the "Patient Information Form" and sign a document giving consent.

### Contact

There is a Technical Secretariat located at the *Centro Superior de Investigación en Salud Publica *(*CSISP*), Avda. Cataluña, 21, 46020 Valencia, Spain; mail: secretariatecnica@escarval.info. Escarval-Risk is one of the studies for a broader CVD research project called ESCARVAL. More information could be found in http://www.escarval.info/.

### Statistical analysis

The main analyses will be conducted according to a statistical plan drafted before completion of data collection. Statistical analyses will address the main study objectives. Accordingly, CV observed risk will be calculated and compared to expected risk according to CV risk scales (Framingham, Score) [6].

For descriptive analysis the cut-points defining control of each risk factor will be taken from the European Guidelines on CVD prevention [6]; for estimates of dyslipidemia control based on LDL and non-HDL cholesterol, we will use the cut-points agreed in the consensus statement from the American Diabetes Association and the American College of Cardiology Foundation [12].

The risk of CVD death will be calculated with the SCORE equation using the SCORE equation for low-risk regions [13]. The risk of CVD morbidity will be calculated with the Framingham equation [14] and other similar scales validated for Spanish population [8,9].

The primary outcome for this study will be the estimation of the probability of CVD in the presence of other competitive events. Cumulative incidence of cardiovascular events will be analyzed by competing risk survival methods, where each subject is at risk of failure from different causes.

Competing risk survival analysis methods will be applied to estimate the cumulative incidence of developing CVD events over time [15]. This method allows for the fact that a patient may experience an event which is different from the event of interest. These events are known as competing risk events, and may preclude the onset of the event of interest, or may modify the probability of the onset of the event of interest. In particular, a hypertensive patient may die without developing any kind of CV disease. In a Kaplan-Meier estimation approach, these persons would be treated as censored and would be eliminated from the risk set. This could lead to misleading results, as it is based on the assumption that censoring is "non-informative", meaning that a censored patient has the same risk of developing CVD as those who have complete follow-up. This is not the case in patients who die before without developing CVD, as they are no longer at risk.

Cumulative incidence functions will be used to compare the observed incidence of events with the predicted ones. The cumulative incidence function regression model of Fine and Gray will be used for multiple regression analyses[16]. Additionally, clinical relevance of different variables will be evaluated using the ARR (Absolute Risk Reduction), RRR (Relative Risk Reduction) and NNT (Number Needed to Treat) and their 95% confidence intervals.

## Discussion

CVD are the leading cause of death despite of the great capacity for prevention so new studies are designed for better understanding these issue and gain insights for designing interventions to overcome the barriers preventing implementation of CVD prevention in clinical practice [17].

ESCARVAL-RISK study can provide relevant information to validate CV risk scales in Mediterranean patients with CV risk factors as hypertension, diabetes and/or dyslipidemia. The information may improve the prediction of CV risk in this specific population. Furthermore, the study can provide a better assessment of CVD risk factors control as well as therapeutic compliance or clinical inertia [18].

The CV risk scales are widely used in primary care to identify high risk patients, and this label (high risk) has implications specially to decide when to initiate or not pharmacological treatment. Nowadays, SCORE risk scale is recommended by European and Spanish Scientific Societies to use in clinical practice [6,19]. It has been published that these widely used scales (Framingham, Score) overestimate high risk in Mediterranean populations [20,21], so we could have many over treated patients. On the other hand, if there are high risk patients that are not identified and not treated, CVD could not be prevented in these patients. So ESCARVAL-RISK will also create opportunities to improve clinical practice in Mediterranean regions.

We had a comprehensive framework for physician sampling in the Valencia Community as all of them are working in the Public Health System that is universal for all citizens. Every physician and nurse has a list of patients (average 1600) for clinical practice. If one patient changes to one physician to another we still have the information for the events although the new physician decides not to participate in the study.

The participation rate among invited clinicians was very high. This high percentage is probably due to both, the support of Valencian Health Authorities and the local leadership of the researchers. The fact of participating in this study (Hawthorne effect) may slightly overestimate the control of CVD risk factors and quality of care in usual clinical practice.

Nevertheless, the large number of practitioners included, the coverage of ABUCASIS electronic system, help for that ESCARVAL-RISK study is likely to provide a good validation of the risk scales for this European Mediterranean population and a comprehensive picture of the status of primary CVD prevention. Blood samples have been analyzed with the same methods in reference hospital laboratories in the same region. The results are automatically sent from the laboratory to the electronic clinical record once validated and signed by the analyst. So all results are included in the clinical record and it does not depend on the criteria of the clinician that could register only the results that were out of range infra estimating the normal values.

Assessment of control of CVD risk factors is based on objective measures (blood pressure readings, anthropometry, and laboratory results) specifically obtained for the ESCARVAL-RISK study. Thus, data quality in the ESCARVAL-RISK study is likely to exceed that of studies relying solely on retrospective data abstraction from clinical records and interviews.

The electronic ABUCASIS system for primary and secondary ambulatory care is an opportunity to initiate new studies about how CV and other diseases are managed in real clinical practice. It permits a new kind of data based in whole population studies and opens a room for new epidemiological studies and at last for improvement clinical practice and for giving best care for patients. Other study has been initiated about osteoporosis prevention using the same design for the validation of a population-based prediction scale for osteoporotic fracture: the ESOSVAL-R study [22].

Finally, the ESCARVAL-RISK study will provide information about the real incidence of CVD in the population with hypertension, diabetes or dyslipidemia of the Region of Valencia (which can be useful for other Mediterranean regions). And also will help knowing the real weight of each risk factor for CVD in this context. Not only will constitute a useful prognostic tool, when further developed with research on cost-utility, it will help establish efficient criteria to identify the level of risk at which treatment should be initiated.

## Abbreviations

Authors CV: Cardiovascular; CVD: Cardiovascular disease; CHD: Coronary Heart Disease; PIN: Personal Identification Number; IDC: International Classification of Diseases; LDL: Low Density Lipoprotein; HDL: High Density Lipoprotein;

## Competing interests

The authors declare that they have no competing interests.

## Authors' contributions

VGG made the first draft of the manuscript in Spanish. DOB and SPF made the second draft and translated to English version. VGG, JR, DOB, JN, VP, JMM, FV, CF are members of the Escarval Scientific Board (ESB) and contributes in several parts of the Study (ABUCASIS modifications, database designs, tuition of participating clinicians). AF analyzed database. MP as Abucasis's general coordinator revised all information concerning Abucasis system. JLT as GAIA's general coordinator revised all information concerning GIAIA prescription database. RDA, RC, as ESB's advisers revised the manuscript. All authors contributed to the writing of the manuscript, corrected draft versions and approved the final manuscript.

## Pre-publication history

The pre-publication history for this paper can be accessed here:

http://www.biomedcentral.com/1471-2458/10/717/prepub
